# Marburg virus-like particles by co-expression of glycoprotein and matrix protein in insect cells induces immune responses in mice

**DOI:** 10.1186/s12985-017-0869-3

**Published:** 2017-10-25

**Authors:** Weiwei Gai, Xuexing Zheng, Chong Wang, Hualei Wang, Yongkun Zhao, Qi Wang, Gary Wong, Weijiao Zhang, Na Feng, Boning Qiu, Hang Chi, Nan Li, Tiecheng Wang, Yuwei Gao, Junjie Shan, Songtao Yang, Xianzhu Xia

**Affiliations:** 1 Department of Preventive Veterinary Medicine, College of Veterinary Medicine, Jilin University, Changchun, China; 2 Key Laboratory of Jilin Province for Zoonosis Prevention and Control, Institute of Military Veterinary, Academy of Military Medical Sciences, Changchun, China; 3 School of Public Health, Shandong University, Jinan, China; 4 State Key Laboratory of Veterinary Biotechnology, Harbin Veterinary Research Institute, Chinese Academy of Agricultural Sciences, Harbin, China; 5 College of Animal Science and Technology, Shihezi University, Shihezi, China; 6 CAS Key Laboratory of Pathogenic Microbiology and Immunology, Institute of Microbiology, Chinese Academy of Sciences, Beijing, China; 7 Institute of Pharmacology and Toxicology, Academy of Military Medical Sciences, Beijing, China; 8 Department of Virology, Institute of Military Veterinary, Academy of Military Medical Sciences, 666 Liuying West Road, Changchun, Jilin 130012 People’s Republic of China

**Keywords:** Marburg virus, Virus-like particle, Adjuvant, Vaccine, Immune response

## Abstract

**Background:**

Marburg virus (MARV) causes severe haemorrhagic fever in humans and nonhuman primates and has a high mortality rate. However, effective drugs or licensed vaccines are not currently available to control the outbreak and spread of this disease.

**Methods:**

In this study, we generated MARV virus-like particles (VLPs) by co-expressing the glycoprotein (GP) and matrix protein (VP40) using the baculovirus expression system. MARV VLPs and three adjuvants, Poria cocos polysaccharide (PCP-II), poly(I:C) and aluminium hydroxide, were evaluated after intramuscular vaccination in mice.

**Results:**

Murine studies demonstrated that vaccination with the MARV VLPs induce neutralizing antibodies and cellar immune responses. MARV VLPs and the PCP-II adjuvant group resulted in high titres of MARV-specific antibodies, activated relatively higher numbers of B cells and T cells in peripheral blood mononuclear cells (PBMCs), and induced greater cytokine secretion from splenocytes than the other adjuvants.

**Conclusion:**

MARV VLPs with the PCP-II adjuvant may constitute an effective vaccination and PCP-II should be further investigated as a novel adjuvant.

## Background

Marburg virus (MARV) belongs to the Filoviridae family, which consists of non-segmented, negative-strand RNA viruses that cause severe haemorrhagic fever with mortality rates up to 90% [1, 2]. The first recognized MARV outbreak occurred in Marburg, Germany in 1967 after the importation of infected monkeys from Uganda [3, 4]. Since then, MARV has caused more than 592 human infections and more than 480 deaths. The most recent outbreak occurred in 2014 in Uganda [5]. Because of the high lethality rates and rapid onset, MARV and other viruses have been actively pursued in the past as potential biological weapons [6].

Currently, vaccination is believed to be the best option for preventing MARV disease. Although effective treatments or licensed vaccines against MARV infection are not currently available, substantial progress has been made in the search for a MARV vaccine over the past several years [7–10]. DNA vaccines, recombinant vesicular stomatitis virus vectored vaccines and virus-like particle (VLP) vaccines have been demonstrated to work as prophylactic vaccines and post-exposure treatments in animal models [11]. VLPs are viral proteins that self-assemble into structures resembling the conformation of the authentic native virus; however, they lack a viral genome. Therefore, VLPs are safe and have been successfully developed into commercialized vaccines or candidate vaccines for porcine circovirus (PCV) type 2, hepatitis B virus (HBV), human papillomavirus (HPV) and human immunodeficiency virus (HIV) [12–15]. Because of the high yield, easy construction and large packaging capacity, insect cell baculovirus expression systems have been commonly used for VLP studies [16, 17]. VLPs are capable of activating cells involved in both innate and adaptive immunity, and they can induce strong humoral and cellular immune responses [18–20].

The MARV genome encodes the following seven structural proteins: nucleoprotein (NP), virion protein (VP) 35, VP40, glycoprotein (GP), VP30, VP24, and RNA-dependent RNA polymerase (L) [2]. GP is the primary antigen for eliciting protective immune responses [21–23]. A previous study showed that the GP and VP40 from MARV assemble into VLPs in mammalian cells, and these VLPs are capable of conferring effective protection as a vaccine against a lethal MARV challenge in mice and inducing both humoral and cellular immune responses [21, 23]. Subsequent studies with VLPs containing MARV GP, VP40 and NP, which were generated using a baculovirus expression system, demonstrated that this combination confers protection in guinea pigs and cynomolgus macaques [7, 24]. Recently, we showed that the co-expression of GP and VP40 in insect cells also led to the efficient assembly and release of VLPs. Electron microscopy findings indicated a similar morphology with wild-type MARV [25].

At present, the trend in vaccine development has shown that antigens often lack sufficient immunogenicity, thus requiring the addition of potent adjuvants [26]. Adjuvants have been traditionally used to increase or modulate the humoral or cellular immune response against a vaccine antigen, and reduce vaccine costs by limiting the amount of required antigen. With advances in vaccine technology, many immune potentiator adjuvants have emerged. Natural polysaccharides have been found to act as immunologic enhancers that can be used as an immunopotentiator for enhancing cellular immunity and promoting antibody production. These polysaccharides are natural, safe and non-residual [27–29]. Poria cocos has a long history of medicinal use in China. Their olysaccharides and derivatives exhibit many beneficial medicinal biological activities, including anticancer, anti-inflammatory, antioxidant and antiviral activities [30–32]. In our previous study, a new polysaccharide (PCP-II) was isolated from the sclerotium of Poria cocos. PCP-II has a molecular weight of 29.0 kDa, and it consists of fucose, mannose, glucose and galactose in a molar ratio of 1.00:1.63:0.16:6.29, respectively. PCP-II stimulated significantly antibody responses and extended the durable immunity to an inactiveted rabies vaccine, H1N1 influenza and HBsAg vaccine [33, 34].

In this study, we generated VLPs from insect cells by co-expressing MARV GP and VP40 proteins using the recombinant baculovirus expression system. To assess whether the MARV VLPs alone or with adjuvant can induce specific antibody and cellular immune responses, we evaluated the immunogenicity of MARV VLPs in a mouse model and the results are presented herein.

## Methods

### Cells and antibodies

Spodoptera frugiperda (Sf9) insect cells were cultured in SF-900II serum-free medium (Life Technologies, San Diego, CA, USA) with 0.5% *v*/v penicillin/streptomycin in suspension. Polyclonal antisera against MARV GP and VP40 proteins were isolated from the mice immunized with GP and VP40 proteins expressed by the prokaryotic expression system, respectively [35–37]. Poly(I:C) (Sigma, USA) and aluminium hydroxide (Alum) (Thermo, USA) were purchased, and the PCP-II polysaccharide was kindly provided by Professor Shan Junjie of the Beijing Institute of Pharmacology and Toxicology.

### Generation of recombinant baculoviruses

The GP and VP40 gene sequences of MARV were obtained from GenBank (Accession No. DQ217792 and KF600645, respectively). The genes were codon-optimized for the highest possible expression levels in insect cells and biochemically synthesized (Sangon Biotech, Shanghai, China). The GP gene was inserted into the pFastBacDual vector (Invitrogen, Carlsbad, CA, USA) under the control of the polyhedron promoter, whereas the VP40 gene was inserted into the pFastBacDual vector under the control of the p10 promoter to generate recombinant plasmid pFastBacDual-G-VP40. pFastBacDual-G-VP40 was then transformed into E. coli DH10Bac competent cells to generate recombinant bacmids. Sf9 cells were transfected with the recombinant bacmids using Lipofectamine 2000 according to the manufacturer’s instructions (Invitrogen). Recombinant baculoviruses were harvested 5 days after transfection and expanded in Sf9 cells to generate virus stocks. Titres of baculovirus stocks were determined using a BacPak Baculovirus Rapid Titre Kit (Clontech, USA).

### Generation of VLPs

Sf9 insect cells were infected with recombinant baculovirus at a multiplicity of infection (MOI) of 2, and MARV VLPs were collected from the culture supernatant at 96 h post-infection. After clarifying the cell debris, the VLPs were concentrated by high-speed centrifugation and further purified through a discontinuous sucrose gradient (20%–60%) as previously described [23]. A visible band between the 40% and 60% sucrose layers was collected, concentrated by centrifugation and then resuspended in phosphate buffered saline (PBS).

### Indirect immunofluorescence assay (IFA)

Sf9 cells were infected with the recombinant baculoviruses for 48 h and then fixed with 80% cold acetone for 30 min at room temperature. Subsequently, the cells were evaluated using mouse polyclonal antibodies against MARV VP40 protein and against MARV GP at a final concentration of 10 μg/mL containing 1% bovine serum albumin for 1 h at room temperature. After three washes with PBS 0.05%Tween (PBST) and blocking with PBST plus 2% BSA for 2 h at room temperature, secondary antibodies (FITC-labelled goat anti-mouse IgG) were added with 0.3% Evans blue for 1 h. The cells were then analysed under a fluorescence microscope.

### Characterization by electron microscopy and western blot

For the electron microscopy analysis, MARV VLPs were applied to a carbon-coated formvar grid, negatively stained with 1% phosphotungstic acid and observed via transmission electron microscopy (TEM). The MARV VLPs were analysed by 10% SDS-PAGE under denaturing conditions, and the proteins were transferred onto a polyvinylidene fluoride (PVDF) membrane (Immobilin-P, Millipore, USA) for the Western blot analysis with mouse polyclonal antibodies against MARV VP40 protein and against MARV GP. Detection was then performed with goat anti-mouse horseradish peroxide-conjugated antibody and enhanced chemiluminescence.

### Immunization studies

The protein concentration of the purified MARV VLPs was analysed using a BCA assay kit (Thermo). Six-to-eight-week-old female BALB/c mice were randomly divided into 5 groups with 20 mice per group and then vaccinated intramuscularly (IM) twice with 10 μg of MARV VLPs alone or mixed with the different studied adjuvants. Immunizations were performed on study days 0 and 14. The control mice were vaccinated with PBS at the same time points. For each vaccine injection, 200 μg of either PCP-II, poly (I:C) or Alum adjuvant was mixed with the MARV VLP vaccine. Blood was collected from mice at days 0, 14 and 28.

### Flow cytometry assays for intracellular cytokine staining (ICS)

Peripheral blood mononuclear cells (PBMCs) were collected from 3 mice from each group at 14 days after the first immunization. Single cell suspensions (1 × 10^6^ cells/mL) were prepared in PBS and stained with anti-mouse CD19 and CD40 antibodies (BD Biosciences, Franklin, TN, USA) to label the B cells and with anti-mouse CD3, CD4 and CD8 monoclonal antibodies (BD Biosciences, USA) to label the T cells. Splenocytes were isolated at two weeks after the second vaccination, and splenocyte suspensions (1 × 10^7^ cells/ml) were stimulated with purified GP (10 μg/ml) associated with a protein transport inhibitor (BD Biosciences). At 6 h post-stimulation, surface staining was performed with anti-CD4 and anti-CD8 monoclonal antibodies for 0.5 h at 4 °C, and then the cells were permeabilized with Cytofix/Cytoperm (BD Biosciences) and stained with anti-IFN-γ and anti-IL-4 monoclonal antibodies (BD Biosciences), respectively, for 0.5 h at 4 °C. All labelled cells were washed twice with PBS, and 20,000 cells were analysed in a LSR-II flow cytometer (BD Biosciences).

### Detecting cytokine secretion after immunization

To detect the levels of IL-2, IL-4, IL-10 and IFN-γ cytokines, splenocytes (2 × 10^6^ cells/ml) were isolated at 14 days after the second vaccination and then suspended in RPMI 1640 Medium containing 10% foetal bovine serum (FBS). The splenocyte suspensions were stimulated with purified GP (10 μg/ml) and incubated for 48 h at 37 °C. The supernatants of stimulated cells were measured using murine IL-2, IL-4, IL-10 and IFN-γ ELISA kits according to the manufacturer’s instructions (Mabtech AB, Sweden).

### IFN-γ and IL-4 enzyme-linked Immunospot (ELISpot) assays

Splenocytes from vaccinated mice were isolated at 2 weeks after the second immunization and stimulated with purified GP (10 μg/ml) for 36 h at 37 °C. Splenocytes producing IFN-γ or IL-4 were quantified with a mouse IFN-γ/IL-4 ELISPOT kit (Mouse IFN-γ/IL-4 ELISPOT kit, Mabtech AB, Stockholm, Sweden) according to the manufacturer’s instructions. Spot-forming cells (SFCs) were counted using an automated ELISpot reader (AID ELISPOT reader-iSpot, AID GmbH, GER).

### Pseudotype titration and neutralisation assay

MARV GP-pseudotyped virus preparation and neutralizing antibody (nAb) titre determination were performed as previously described [38]. Briefly, 10 μg of pCAGG-MARV-G and 10 μg of pNL4–3.luc.RE were co-transfected into 293 T cells cultured in a 10-cm dish. The supernatant was harvested after 36 h, centrifuged to remove cell debris, and stored at −80 °C. For the titration assays, 10-fold serial dilutions of pseudotyped virus were incubated with 2 × 10^4^ Vero cells for 48 h. The luciferase activity was measured using a multi-function microplate reader (Infinite M200, Tecan Austria GmbH, Austria).

For the neutralization assay, two-fold serial dilutions ranging from 1:10 to 1:5120 were prepared in quadruplicate. Diluted serum samples were added to 100 x TCID_50_ of pseudotyped virus (prepared in an equal volume as the sera) and incubated at 37 °C for 30 min before addition to the Vero cells. After incubation for 4 h, the medium was replaced with DMEM containing 10% FBS. The plates were incubated for 48 h at 37 °C, and the luciferase activity was measured using Infinite M200. The 50% neutralization dose (ND_50_) was calculated using GraphPad Prism.

### Determination of antibody titres

MARV-specific antibodies were determined by indirect ELISA. Briefly, the assays were performed in 96-well polystyrene microtiter plates (Corning Costar, USA) that were pre-coated with purified GP proteins at a concentration of 1 μg/ml overnight at 4 °C and blocked for 2 h at 37 °C. Serial dilutions of serum samples were incubated at 37 °C for 2 h and HRP-conjugated goat anti-mouse IgG polyclonal antibody (Abcam, UK) was diluted to 1:20,000 and incubated at 37 °C for 1 h. The substrate TMB (Sigma, USA) was added to each well, incubated for 0.5 h and stopped with 2 M H_2_SO_4_. Infinite M200 was used to measure the optical density at 450 nm.

### Statistical analysis

Data are presented as the mean ± standard error of the mean (SEM). Statistical analyses were performed using SPSS 13.0 software (SPSS Inc., Chicago, IL, USA) to determine statistically significant differences in the generated data via a one-way analysis of variance (ANOVA). Significant differences were determined by a Kaplan–Meier analysis. The results were considered significant at *p* < 0.05 and very significant at *p* < 0.01.

## Results

### Generation of recombinant baculovirus

**Fig. 1:**
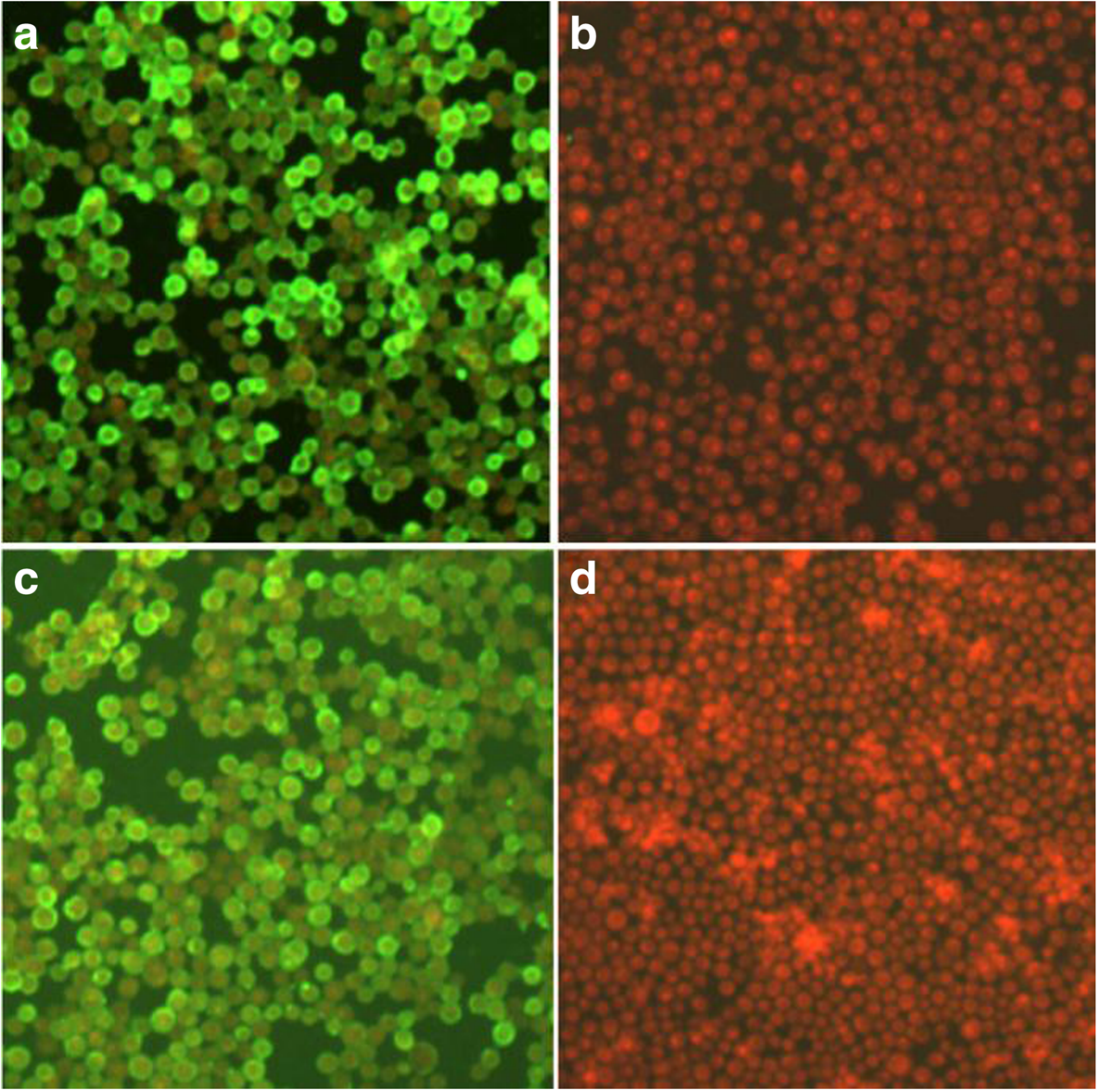
Detection of GP and VP40 expression in the Sf9 cells. Sf9 cells were infected with the recombinant baculoviruses in (**a**) and (**c**). Additionally, mock-infected Sf9 cells were treated with PBS in panels (**b**) and (**d**). The IFA assay was performed with murine anti-MARV GP polyclonal antibody (**a** and **b**) or murine anti-MARV VP40 polyclonal antibody (**c** and **d**) (Magnification of microscopy images, ×200)

The GP and VP40 genes based on the Musoke strain of MARV were cloned into the pFastBacDual vector. The recombinant plasmid was transfected into Sf9 cells to obtain recombinant baculovirus. The expression of GP and VP40 of the recombinant baculovirus was confirmed by IFA. As expected, the results demonstrated the expression of GP and VP40 proteins (Fig. 1).

### Production and identification of MARV VLPs

**Fig. 2:**
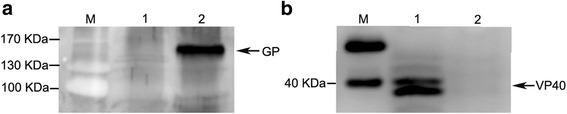
Western blot analysis of GP and VP40 protein expression in the MARV VLPs. 10 μg of MARV VLPs by co-expression of GP and VP40 were mixed with reducing (with β-mercaptoethanol) protein sample buffer, heated at 95 °C for 5 min, and then subjected to 10% SDS-PAGE with different gels. Two different gels were transferred onto a polyvinylidene fluoride (PVDF) membrane for the Western blot analysis, respectively. **a** GP was incubated with mouse anti-MARV GP polyclonal antibody (control, lane 1; MARV VLPs, lane 2) and the molecular weight were approximately 150KD. **b** VP40 proteins was incubated with mouse anti-MARV VP40 polyclonal antibody (MARV VLPs, lane 1; control, lane 2) and the molecular weight were approximately 38KD

**Fig. 3:**
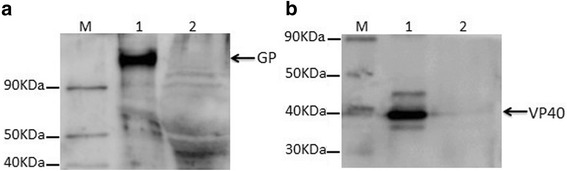
Western blot analysis of GP and VP40 protein expression in the purified MARV VLPs. 10 μg of the purified MARV VLPs were mixed with reducing (with β-mercaptoethanol) protein sample buffer, heated at 95 °C for 5 min, and then subjected to 10% SDS-PAGE with different gels. Two different gels were transferred onto a polyvinylidene fluoride (PVDF) membrane for the Western blot analysis, respectively. **a** GP was incubated with mouse anti-MARV GP polyclonal antibody (purified MARV VLPs, lane 1; control, lane 2). **b** VP40 proteins was incubated with mouse anti-MARV VP40 polyclonal antibody (purified MARV VLPs, lane 1; control, lane 2)

**Fig. 4:**
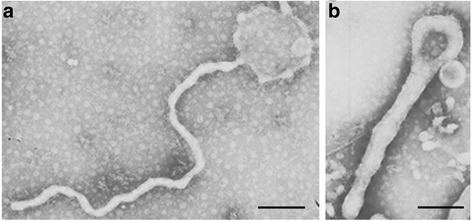
Electron microscopy of the MARV VLPs. At 96 h post-infection, the mixture of supernatant and cells was collected. The samples were stained with 1% sodium phosphotungstate and then observed via transmission electron microscopy. **a** and **b** showed long fiaments shaped in several different form to authentic MARV. MARV VLPs had a diameter of approximately 50 nm (**a**) or 100 nm (**b**). Bar = 200 nm

Sf9 cells were infected with recombinant baculovirus at a MOI of 2, and the mixture of supernatant and cells was analysed via Western blot (Fig. 2) and TEM (Fig. 4). MARV VLPs produced by infected Sf9 cells and purified by sucrose gradient was analysed with Western blot. As shown in Fig. 2a and 3a, a protein band with a molecular weight of 150 KD corresponding to GP was detected in the MARV VLPs. VP40 proteins were also readily detected in the VP40-GP VLPs at a molecular weight of 38 kD (Fig. 2b and 3b). However, specific bands of GP and VP40 were not detected in the control sample lane. The generated VLPs resemble those of native MARV virions, and the diameters were approximately 50~100 nm (Fig. 4).

### Antibody responses induced by MARV VLPs with adjuvant

**Fig. 5:**
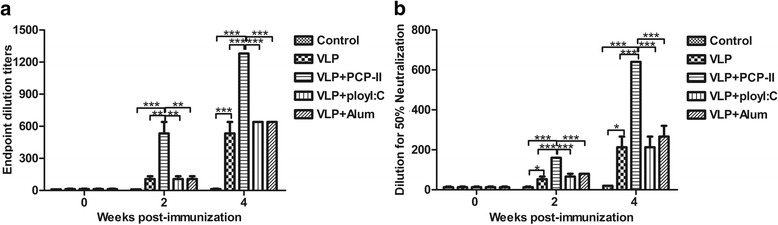
Serum antibody responses to the MARV VLPs. **a** Analysis of the antibody responses by ELISA. Serum samples were obtained 2 weeks after each vaccination, and the total serum antibodies were assayed for MARV GP-specific antibodies using ELISA, with purified GP as the coating antigen. Antibody titres were measured for individual mice, and the results are graphed as the geometric mean titre for each group (*n* = 5). **b** Neutralization of MARV GP-pseudotyped virus. Serum samples were incubated with the MARV GP-pseudotyped virus in Vero cells. The titres were shown as the highest serum dilutions that reduced 50% of the luciferase activity of the wells (NT_50_) and are expressed as the mean ± SD. Statistical significance was analysed using a one-way ANOVA (*p < 0.05, **p < 0.01, and ***p < 0.001)

To evaluate the immunogenicity of the MARV VLPs, the serum IgG concentrations of the immunized mice were determined via ELISA at day 14 (2 weeks after the 1st immunization). All mice immunized with the MARV VLPs had strong humoral responses compared with those observed in the PBS group, and the PCP-II group had higher IgG titres than the animals administered the MARV VLPs alone (*p* < 0.001) (Fig. 5a). Mice immunized with PCP-II as an adjuvant had higher responses (endpoint titre of 1:1280) than those vaccinated with the poly (I:C) or Alum adjuvant (*p* < 0.01) (Fig. 5a). Animals vaccinated with the MARV VLPs mixed with the poly (I:C) or Alum adjuvant had similar responses to the GP antigens (endpoint titre of 1:640) (Fig. 5a).

The nAb titres of all mice immunized with the MARV VLPs were higher than that of the PBS group (Fig. 5b) at day 14 (2 weeks after the 1st immunization). Furthermore, mice vaccinated with PCP-II as an adjuvant had higher nAb titres than those vaccinated with the poly (I:C) or Alum adjuvant (*p* < 0.01). The nAb titres of all mice vaccinated with the MARV VLPs were higher than those of the PBS group, and the nAb titres of the PCP-II group were determined up to a dilution of 1:640 after the second immunization (Fig. 5b) at day 28 (2 weeks after the 2nd immunization). Mice vaccinated with PCP-II as an adjuvant had higher nAb titres than those vaccinated with the MARV VLPs or poly(I:C) (endpoint titre of 1:200) (*p* < 0.001) or Alum (endpoint titre of 1:250) (*p* < 0.001). The nAb titre of the control PBS group was below the limit of detection (endpoint titre of 1:40).

### Antigen-specific cellular immune responses

**Fig. 6:**
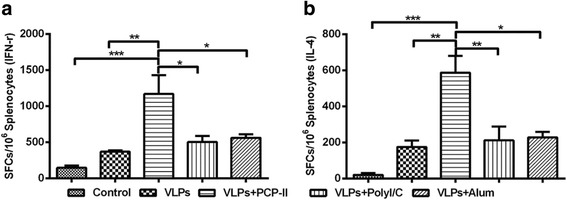
ELISpot analysis of IFN-γ and IL-4 secretion from splenocytes. Spleens were collected from 3 mice per group at 14 days after the second vaccination, and splenocytes were assayed by the ELISpot and ICS assays. SFCs secreting IL-4 (**a**) and IFN-γ (**b**) were measured using ELISpot. The data represent the mean ± SD of SFCs per million splenocytes from 3 mice in each group, and statistical significance was analysed using a one-way ANOVA (*p < 0.05, **p < 0.01, and ***p < 0.001)

To assess the T-cell responses in mice following vaccination, we used an ELISpot assay to detect IFN-γ and IL-4 secretion from mouse splenocytes. Higher numbers of SFCs were observed in the mice injected with PCP-II than in the mice from the other groups (Fig. 6a and b). Also, the level of IFN-γ or IL4 secretion from all mice vaccinated with the MARV VLPs was higher than that observed in the control mice. For IFN-γ, the PCP-II group had more than 1000 SFCs, whereas the other groups had approximately 100~500 SFCs. For IL-4, the PCP-II group had more than 500 SFCs, whereas the other groups had approximately 20~200 SFCs. Overall, these results suggest that the ability of PCP-II to boost cell-mediated immune responses to a MARV VLP vaccine is superior to that of poly(I:C) and Alum.

**Fig. 7:**
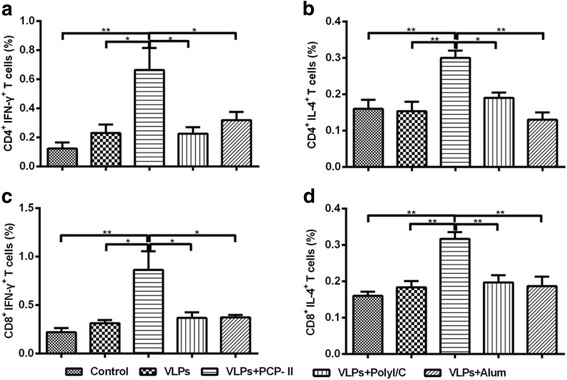
ICS assays for antigen-specific CD4^+^ and CD8^+^ T-cell secretion of IFN-γ and IL-4 from splenocytes. MARV-specific CD4^+^ and CD8^+^ T cells were measured via ICS assays. Spleens were prepared from 3 mice per group and stained with mouse antibodies against CD4, CD8, IFN-γ and IL-4. CD4^+^ cells secreting IL-4 (**a**) or IFN-γ (**b**) and CD8^+^ cells secreting IL-4 (**c**) or IFN-γ (**d**) are shown. The data represent the mean ± SD, and statistical significance was analysed using a one-way ANOVA (*p < 0.05, **p < 0.01, and ***p < 0.001)

To further characterize the T-cell response induced by the MARV VLPs, we evaluated the ability of MARV VLPs plus these adjuvants to induce IFN-γ and IL-4 secretion from CD4^+^ and CD8^+^ T cells with an intracellular cytokine staining (ICS) assay. Compared with the control group, MARV VLPs with PCP-II induced higher numbers of IFN-γ-secreting CD4^+^ or CD8^+^ T cells (*p* < 0.01) (Fig. 7a and c). The MARV VLPs with PCP-II induced higher numbers of IFN-γ-secreting CD4^+^ or CD8^+^ T cells than the other adjuvant groups (*p* < 0.05) (Fig. 7a and c). Similar results were observed for IL-4 secreting CD4^+^ and CD8^+^ T cells (Fig. 7b and d). The MARV VLPs with PCP-II induced relatively higher numbers of IL-4-secreting CD4^+^ or CD8^+^ T cells than the other adjuvant groups (*p* < 0.05) (Fig. 7b and d). Statistically significant differences were not observed between the poly(I:C) or Alum group and the VLP-only group. These data demonstrated that mice immunized with the MARV VLPs containing PCP-II had a significantly enhanced IFN-γ- or IL-4-secreting CD4^+^ and CD8^+^ T-cell response.

### Enhancing effect of PCP on B cell and T cell recruitment in the blood

**Fig. 8:**
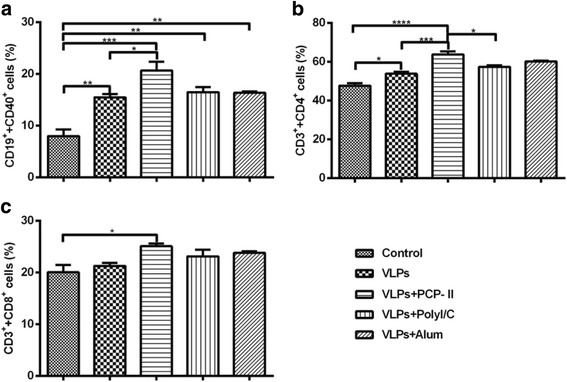
Flow cytometry analysis of T and B cells isolated from the PBMCs of mice. PBMCs were isolated from 3 mice in each group after the first immunization, and they were identified by the following markers: CD19^+^CD40^+^ B cells or CD3^+^CD4^+^CD8^+^ T cells. The double-positive CD19^+^CD40^+^ (**a**), CD3^+^CD4^+^ (**b**) and CD3^+^CD8^+^ (**c**) cells were plotted. Representative data are shown as the mean ± SEM, and statistical significance was analysed using a one-way ANOVA (*p < 0.05, **p < 0.01, ***p < 0.001, and ****p < 0.0001)

To **i**nvestigate whether PCP-II activates more B and T cells in mice than other adjuvants, lymphocytes isolated from PBMCs were collected for T-cell and B-cell isolation. Fig. 8a shows the B-cell activation (CD19^+^CD40^+^), and Fig. 8b and c show the T-cell recruitment (CD3^+^CD4^+^ and CD3^+^CD8^+^) in blood. PCP-II activated the highest numbers of B and T cells of all groups.

### Detection of splenocyte cytokine secretion

**Fig. 9:**
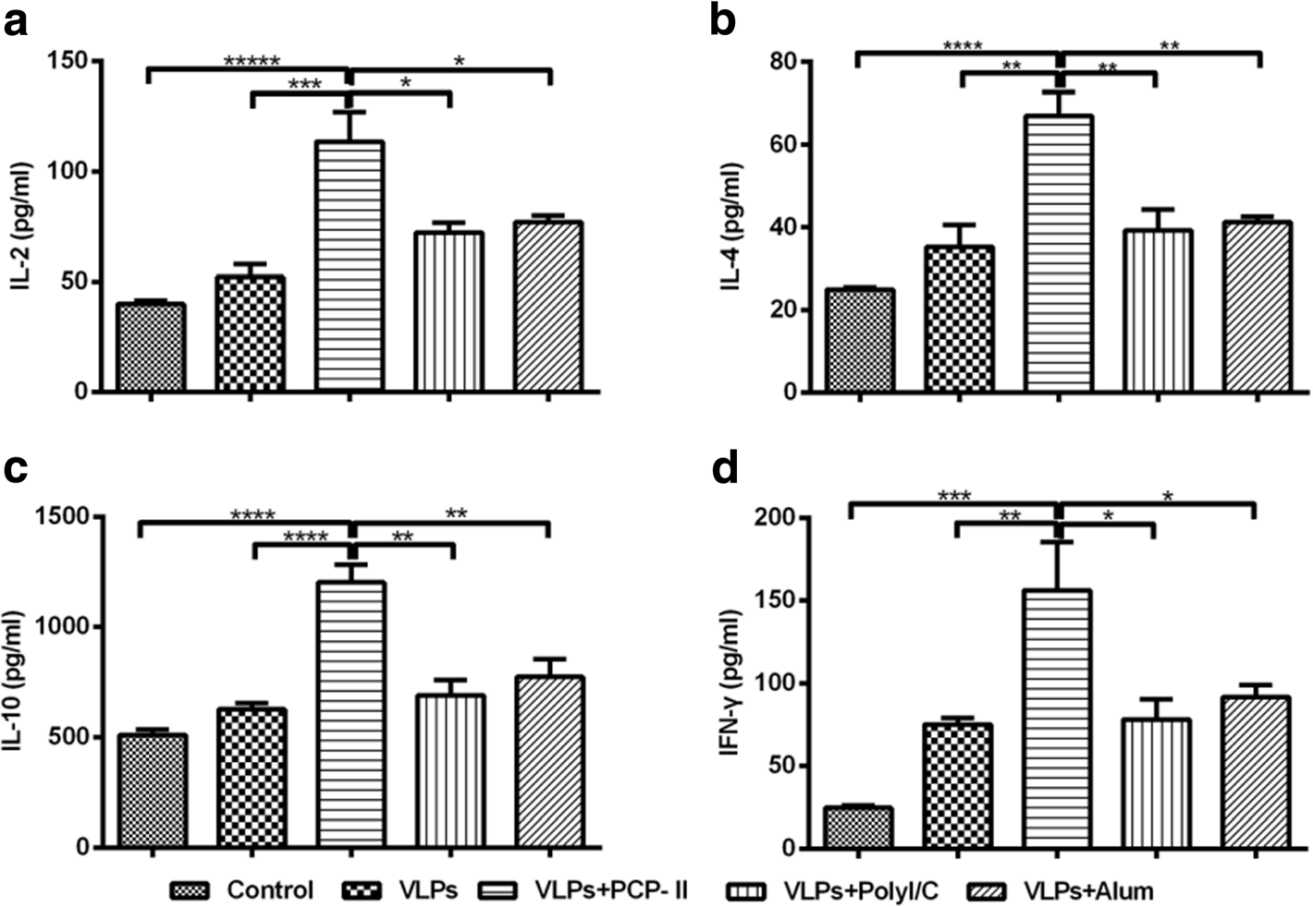
ELISA results showing the quantities of IL-2, IL-4, IL-10 and IFN-γ secreted by splenocytes. Splenocytes were prepared from 3 mice per group at 2 weeks after the second vaccination. Cell-free supernatants were harvested at 48 h after incubation and measured to determine the levels (pg/ml) of IL-2 (**a**), IL-4 (**b**), IL-10 (**c**) and IFN-γ (**d**) via ELISA in duplicate. Representative data are shown as the mean ± SD of 3 mice per group and were analysed using a one-way ANOVA (*p < 0.05, **p < 0.01, and ***p < 0.001)

The cytokine levels were measured in duplicate using commercial ELISA kits for IL-2, IL-4, IL-10 and IFN-γ The levels of IL-2, IL-4, IL-10 and IFN-γ secreted from the splenocytes isolated from mice in the MARV VLPs groups were higher than those in the control group (Fig. 9a–d). The levels of IL-2, IL-4,IL-10 and IFN-γ in the PCP-II group were significantly higher than those in the other adjuvant groups (*p* < 0.05 or *p* < 0.01). The secretion response of IFN-r and IL-2 was associated with a Th1 profile, whereas the secretion responses of IL-4 and IL-10 were associated with a Th2 immune response.

## Discussion

In this study, we generated the typical MARV VLPs by co-expression of just two viral genes, GP and VP40, in insect cells. Compared to co-infection and three viral genes to form VLPs, this kind of program of co-expression of GP and VP40 could improve the level of VLPs expression and enhance packaging efficiency. Due to its multiple culture advantages, the insect cell/baculovirus expression vector system has been a powerful platform for the production of VLPs, including the ability to reach high cell densities in suspension cultures and the high protein expression levels provided with eukaryotic post-translational processing and so on [39].

Adjuvants are compounds that can increase or modulate the intrinsic immunogenicity of an antigen and elicit stronger immune responses. Each adjuvant generates a characteristics immune response profile. And different adjuvants are suitable for different antigens. However, Alum, which is a widely used adjuvant, may cause side effects in certain cases, and its contribution to the induction of early antibody responses is limited [40–42]. Poly(I:C), a synthetic double-stranded RNA, has been demonstrated as a potent adjuvant with the ability to enhance the host’s innate and adaptive immune responses [43–45]. Polysaccharides are emerging as a new type of adjuvant that can be used to stimulate a rapid immune response; thus, they serve as a highly valuable addition for boosting vaccine immunogenicity [46].

RIBI and QS-21 adjuvants are used in many MARV VLPs studies [7, 17, 47, 48]. In this study, the immunogenicity of the MARV VLPs alone or mixed with poly(I:C), Alum or PCP-II as a vaccine was evaluated in mice. Our results showed that vaccination with MARV VLPs stimulated robust immune responses and cellar immune responses in mice. The addition of PCP-II significantly enhanced the specific antibody responses and nAb titres of the MARV VLPs compared with those of the other adjuvants or MARV VLPs alone in mice for the first immunization. Notably, after the second immunization, the mice in the PCP-II group still produced higher levels of nAbs than the other adjuvant groups, and they had the highest overall levels of antibodies at 28 days after immunization. Compared with the mice in the other adjuvant groups, the mice in the PCP-II group induced higher levels of lgG after the second vaccination.

The administration of vaccines composed of MARV VLPs adjuvanted with PCP-II as the adjuvant resulted in a significant increase in the secretion of IFN-γ and IL-2 (Th1-type related cytokines) and IL-4 and IL-10 (Th2-type related cytokines) in our study. Splenocytes from mice immunized with the MARV VLPs also produced stronger IFN-γ and IL-4 responses, which stimulate both arms of the adaptive immune response. Our results showed that the MARV VLPs with PCP-II can boost immune responses through the Th1 and Th2 pathways and indicated that higher numbers of activated T and B lymphocytes circulate in the blood after vaccination with this adjuvant combination than those in mice administered other adjuvants. In turn, the enhanced immunity may also have resulted in a higher and broader serum antibody subclass response.

T lymphocytes are divided into CD4^+^ and CD8^+^ T cells, which mediate antigen-specific cellular immune responses. To further analyse the capacities of these adjuvants to induce IFN-γ and IL-4 secretion from CD4^+^ and CD8^+^ T cells, an ICS assay was performed. The results of the ICS assay showed that the PCP-II adjuvant activates a relatively higher number of IFN-γ- or IL-4-secreting CD4^+^ T cells. IFN-γ secreted by Th1 cells activates macrophages, thereby increasing the killing of phagocytosed microbes. However, IL-4 produced by Th2 cells drives the maturation of B cells into plasma cells, thereby resulting in antibody production, isotype-switching and affinity maturation. Our results indicate that PCP-II acts as an adjuvant that stimulates stronger Th1 and Th2 type responses, therefore, it is superior to both the poly(I:C) and Alum adjuvants.

Both specific humoral and cell-mediated immunity is essential for effective vaccination against many pathogens. In the present study, PCP-II may be more effective at inducing stronger humoral and cellular immunity than other adjuvants when used in conjunction with a MARV VLPs vaccine. Due to the lack of an animal model or current animal models are too costly to obtain, we first test our vaccine candidate and regimens for immunogenicity in mice model. If possible, we will perform challenge experiments to confirm that the enhanced immunity leads to better protection, to advance the best-performing candidate into a suitable animal model for MARV, and to compare PCP-II with RIBI and QS-21 in future studies.

## Conclusion

In summary, PCP-II adjuvanted MARV VLPs is a promising vaccine regimens for the next stage of pre-clinical testing in larger animal models, and the dosage, route of administration and intervals of immunization will need to be further evaluated and optimized to maximize chances of a successful vaccination. Our work highlights the potential for using PCP-II to develop a MARV VLP vaccine for use in humans.

## Abbreviations

ANOVA: A one-way analysis of variance
ELISpot: Enzyme-Linked Immunospot
FBS: Foetal bovine serum
HBV: Hepatitis B virus
HIV: Human immunodeficiency virus
HPV: Human papillomavirus
ICS: Intracellular cytokine staining
IM: Intramuscularly
MARV: Marburg virus
NAb: Neutralizing antibody
PBMCs: Peripheral blood mononuclear cells
PCP: Poria cocos polysaccharide
PCV: Porcine circovirus
PVDF: Polyvinylidene fluoride
SEM: Standard error of the mean
Sf9: *Spodoptera frugiperda*
TEM: Transmission electron microscopy
VLPs: Virus-like particles
